# Dysphagia After Partial Horizontal Supracricoid Laryngectomy: A Close Look

**DOI:** 10.7759/cureus.62124

**Published:** 2024-06-11

**Authors:** Gonçalo Caetano, Filipa Morgado, Joana Póvoa, Francisco Branquinho

**Affiliations:** 1 Otolaryngology - Head and Neck Surgery, Hospital da Senhora da Oliveira, Guimarães, PRT; 2 Otolaryngology - Head and Neck Surgery, Hospital Beatriz Ângelo, Lisboa, PRT; 3 Otolaryngology - Head and Neck Surgery, Instituto Português de Oncologia de Coimbra Francisco Gentil, Coimbra, PRT

**Keywords:** dysphagia, rehabilitation, osteophyte, swallowing, laryngectomy

## Abstract

Partial horizontal supracricoid laryngectomy (SCPL) with cricohyoidoepiglottopexy (CHEP) is a conservative surgical alternative for laryngeal cancer in the glottic or supraglottic region. Dysphagia and aspiration are frequently reported consequences of this surgery.

We describe the case of a 72-year-old male patient diagnosed with squamous cell carcinoma of the larynx (T2N0M0), who underwent SCPL with CHEP reconstruction. The patient was initially fed through a nasogastric tube post-surgery, later replaced by a percutaneous endoscopic gastrostomy (PEG) tube. Swallowing evaluations were periodically conducted in collaboration with a speech therapist using fiberoptic endoscopic evaluation of swallowing (FEES) and videofluoroscopic swallowing study (VFSS). In FEES assessments, the patient consistently presented with laryngeal penetration and possible tracheal aspiration. These findings were confirmed by VFSS. Additionally, a narrowing of the initial segment of the cervical esophagus was observed, caused by a large osteophyte in the anterior region of the C5 vertebral body, compromising the passage of the bolus, and leading to its accumulation above the upper esophageal sphincter and subsequent entry into the airway. Rehabilitation exercises for swallowing were recommended, maintaining an exclusive PEG diet. Three months after rehabilitation, a follow-up VFSS revealed that, for pasty consistency, the accumulation of the bolus above the cervical osteophyte was resolved with multiple swallows, without evidence of penetration or aspiration. Thus, it was possible to introduce oral intake of pasty consistency.

Considering the anatomical and physiological complexity of swallowing, along with patient-specific characteristics, predicting the rehabilitation time for reconstructive laryngeal surgery is challenging. This case emphasizes the importance of a collaborative evaluation involving otorhinolaryngologists, speech therapists, and radiologists in studying dysphagia in patients undergoing conservative laryngeal surgeries to adapt and personalize rehabilitation.

## Introduction

Partial horizontal supracricoid laryngectomy (SCPL) with cricohyoidoepiglottopexy (CHEP) is a conservative surgical alternative for laryngeal cancer in the glottic or supraglottic region. Dysphagia and aspiration are frequently reported consequences of this surgery [1,2].

## Case presentation

**Figure 1:**
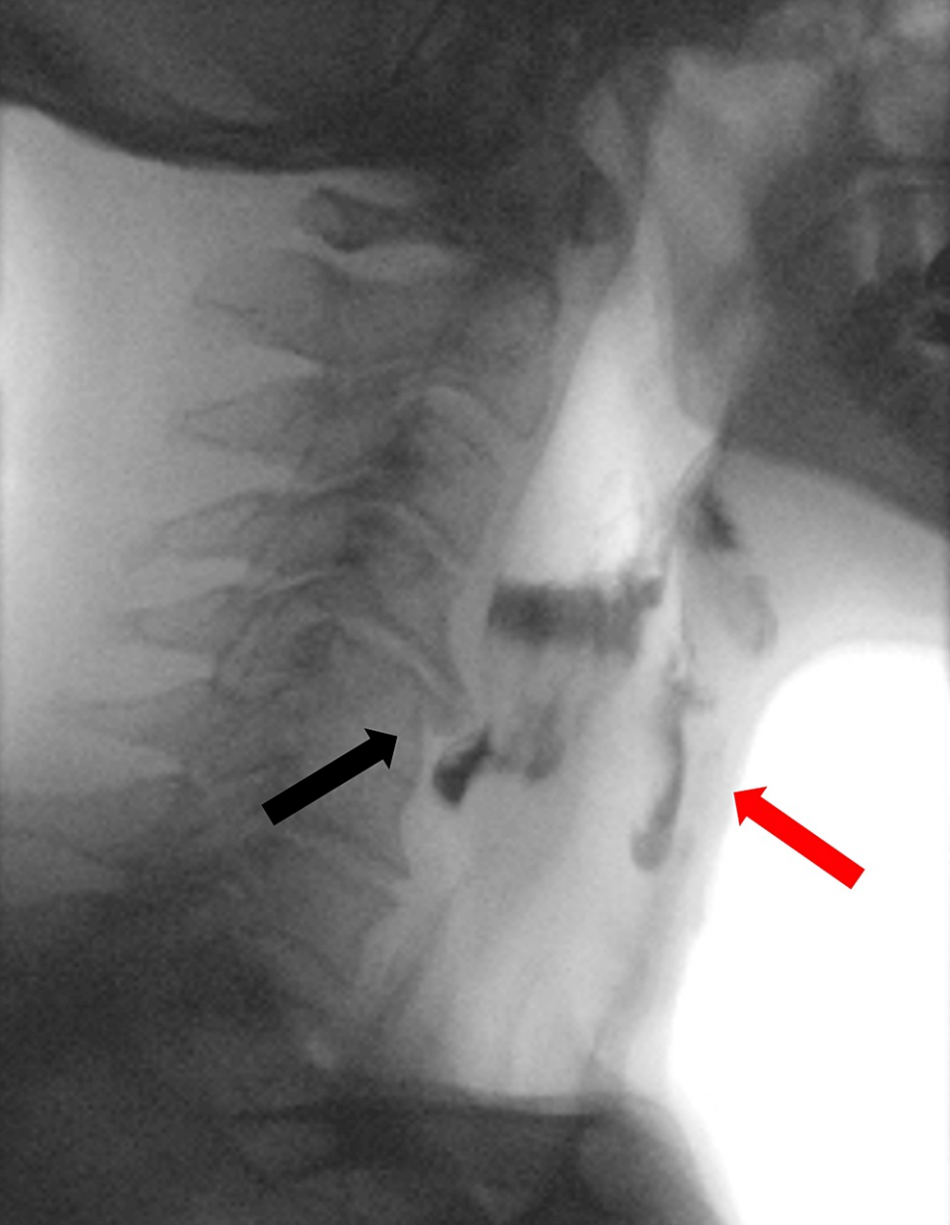
Videofluoroscopic swallowing study. Large osteophyte in the anterior region of the C5 vertebral body (black arrow), compromising the passage of the bolus, leading to its accumulation above the upper esophageal sphincter and subsequent entry into the airway (red arrow).

A 72-year-old male patient diagnosed with squamous cell carcinoma of the larynx (T2N0M0) underwent SCPL with CHEP reconstruction, right cervical lymph node dissection (areas II-IV), and tracheostomy (temporary, currently closed). The patient was initially fed through a nasogastric tube post-laryngeal surgery, which was later replaced by a percutaneous endoscopic gastrostomy (PEG) tube because the patient did not tolerate the nasogastric tube. Swallowing evaluations were periodically conducted in collaboration with a speech therapist using fiberoptic endoscopic evaluation of swallowing (FEES) and videofluoroscopic swallowing study (VFSS). In FEES assessments for liquid and pasty consistencies, the patient consistently triggered coughing after swallowing, with residue present in the hypopharynx and vallecula, along with laryngeal penetration and possible tracheal aspiration. This evaluation was complemented by VFSS, confirming laryngeal penetration and tracheal aspiration. Additionally, a narrowing of the initial segment of the cervical esophagus was observed, caused by a large osteophyte in the anterior region of the C5 vertebral body, compromising the passage of the bolus, and leading to its accumulation above the upper esophageal sphincter and subsequent entry into the airway (Figure 1). Rehabilitation exercises for swallowing were recommended while maintaining an exclusive PEG diet, relaxation and mobilization of the neck and shoulder girdle, supraglottic swallowing in a normal position and with head flexion, Shaker exercise, and vocal exercises with laryngeal elevation and sustainment.

The patient was informed about the reserved prognosis to manage expectations. Three months after rehabilitation, a follow-up VFSS was conducted, with findings of laryngeal penetration for honey consistency. However, for pasty consistency, the accumulation of the bolus above the cervical osteophyte was resolved with multiple swallows, without evidence of penetration or aspiration. Thus, it was possible to introduce oral intake of pasty consistency. To this day, further diet progression has not been achieved.

## Discussion

The presented case underscores the challenges associated with dysphagia following partial horizontal SCPL with CHEP reconstruction. Despite the anatomical and physiological complexities involved in swallowing, this case demonstrates the potential for personalized rehabilitation and the importance of collaborative evaluation involving otorhinolaryngologists, speech therapists, and radiologists. By utilizing a combination of FEES and VFSS, along with tailored rehabilitation exercises, it was possible to improve swallowing function in our patient [3]. This case emphasizes the need for a multidisciplinary approach to adapt and personalize rehabilitation strategies for patients undergoing conservative laryngeal surgeries [4].

## Conclusions

Predicting rehabilitation time for reconstructive laryngeal surgery is challenging due to the complex anatomy and physiology of swallowing and individual patient factors. This case underscores the need for a collaborative evaluation by otorhinolaryngologists, speech therapists, and radiologists. Such teamwork is essential for studying dysphagia in patients undergoing conservative laryngeal surgeries and tailoring their rehabilitation.
